# Give birth in rural hometowns or urban areas? Place of delivery among rural migrant working mothers in the Pearl river delta, China

**DOI:** 10.1186/s12884-025-07692-z

**Published:** 2025-05-29

**Authors:** Chunlan Guo, Yun Li, Yunan Chen, Yang Lyu, Shanshan Wang, Zhenxiang Zhang, Dong Dong

**Affiliations:** 1 Department of Geography and Resource Management, The Chinese University of Hong Kong, Sha Tin, Hong Kong SAR China; 2 Shenzhen Research Institute, The Chinese University of Hong Kong, Shenzhen, China; 3 School of Architecture and Urban Planning, Shenzhen University, Shenzhen, China; 4 School of Nursing, The Hong Kong Polytechnic University, Hung Hom, Hong Kong SAR China; 5 School of Nursing and Health, Zhengzhou University, Zhengzhou, China; 6 The Jockey Club School of Public Health and Primary Care, The Chinese University of Hong Kong, Sha Tin, Hong Kong SAR China

**Keywords:** Rural-urban migration, Working mothers, Childbirth, Place of delivery, The Pearl river delta

## Abstract

**Background:**

In China, the participation of rural mothers in urban labor markets is on the rise, but there’s limited knowledge about the place of delivery among them. Why do certain rural migrant working mothers choose to return to their rural hometowns for childbirth, while others opt to deliver in urban areas?

**Methods:**

This study analyzed the data of 1852 rural migrant working mothers collected from the China Migrant Dynamic Survey in the Pearl River Delta (PRD). These mothers, each with at least one child under the age of 18, had left the location of their agricultural hukou for employment or business in the PRD.

**Results:**

The results indicated that 63.7% of the surveyed mothers returned to rural hometowns for childbirth, with the remaining 36.3% choosing to give birth in urban areas. Factors that positively influenced their decision to deliver in urban areas included self-employment, postsecondary education, higher household income, longer migration duration and exposure to received health education regarding reproduction, contraception/eugenics, and nutrition. On the other hand, inter-provincial migration and earlier birth year negatively influenced rural migrant working mothers’ giving birth in urban areas.

**Conclusion:**

This study offers insights into childbirth strategies adopted by rural migrant working mothers that can shape future policy studies addressing internal rural-to-urban migration, women, maternal health and childcare services.

## Introduction

The decision of where to deliver a baby can have life-altering consequences for both the mother and child [1, 2]. In the topic of place of delivery, existing research primarily concentrates on the selection of the place, typically evaluating the options between home and hospital settings [e.g., 3, 4], selection of a specific hospital and its determinants [e.g., 5, 6]. The widespread adoption of hospital births since the 1960s has played a significant role in reducing stillbirths, neonatal deaths and preventing various child disabilities [3]. However, in underdeveloped countries, limited access to professional obstetrics care forces women to have limited choices, resulting in a high percentage of home deliveries [4–6]. This situation of unmet obstetric needs contributes significantly to the high rates of maternal mortality observed in these countries [7]. In sub-Saharan Africa, several key barriers to accessing and utilizing emergency obstetric care have been identified across different layers [8]: the first layer encompasses barriers such as younger age, illiteracy, lower income, unemployment and cultural beliefs [9]; the second layer involves physical infrastructure and transportation challenges, such as poorly designed roads, lack of vehicles, transportation costs and distance from healthcare facilities [9, 10]; and the third layer comprises barriers within the healthcare systems itself, including the absence of emergency obstetric care services and supplies, shortage of trained staff, inadequate management of emergency obstetric care provision, high cost of services, long waiting times, deficient referral practices, and inadequate coordination among staff [8].

On the other hand, even in developed countries where obstetrics services are more readily available, there remain concerns about meeting women’s expectations and ensuring their satisfaction with obstetrics services. In Denmark, women’s complaints concerning obstetric care differed from other types of healthcare services [11]. Specifically, women who received obstetric care expressed a larger number of issues per complaint compared to other healthcare services. Furthermore, they were more likely to raise concerns related to relational aspects of care, as evidenced by the number of complaints regarding staff shortage being four times higher in the obstetric care group [11]. Indeed, selecting a hospital for childbirth is an integral part of the preparation process for women expecting to give birth to a child. When deciding the place of delivery, women usually prioritize the safety of the mother and child. In Poland, for example, most mothers, both from the city and the countryside do not choose the nearest hospital since they are concerned about the availability of a neonatal intensive care unit at the chosen delivery place in the case of health problems with the newborn baby [12]. The information they received about the options and choices of hospitals available to them, previous birth experiences, perceptions of family, friends and healthcare professionals, and women’s beliefs about risk and safety all have significant influences on where to give birth [13]. Additionally, there is a growing interest in alternative birthing settings such as home births and birthing centers, in developed countries [14, 15]. Studies have shown that planned home births can be as safe as planned hospital births for low-risk women without medical complications in countries with well-functioning healthcare systems [16–18].

Migrant mothers may confront more different choices and challenges than non-migrants when they are making the decision of where to deliver a baby. Research indicates that migrant women in high-income countries, especially those from non-English speaking backgrounds, are at a higher risk of adverse birth outcomes such as stillbirth, neonatal mortality, and maternal death, than the local-born women [19]. Moreover, in developing countries such as Viet Nam, the likelihood of not giving birth in a healthcare facility (e.g., hospital) for ethnic minority women residing in rural areas exhibited a substantial increase and this risk was approximately 20 times higher compared to women of the majority ethnicity [20]. Migrant mothers may encounter difficulties related to language barriers and cultural adaptation, affecting their access to healthcare, educational resources and community support for themselves and their children [21, 22]. Moreover, the challenges faced by migrant mothers extend to financial stability, as they may struggle to afford traveling costs for healthcare services, leading to delays in seeking necessary care [23]. The challenges faced by migrant mothers highlight the importance of understanding their unique circumstances and providing appropriate support to ensure positive maternal and child health outcomes.

In China, despite a significant increase in the total expenditure for facility-based deliveries (both vaginal and caesarean), there has been a substantial rise in the number of women opting for childbirth at healthcare facilities from 55% in 1996 to 90% in 2007 [24]. During almost the same period,, neonatal mortality in China experienced a 62% reduction between 1996 and 2008 [25]. By 2023, the proportion of facility-based deliveries in China had reached a remarkable 99.95%, with 99.97% in urban areas and 99.91% in rural counties [26]. This suggests that almost all mothers in China are now choosing facility-based deliveries. The development of the “world factory” in China since economic reform in 1978 has created a demand for a large number of laborers. Notably, many of the export-oriented industries favor female workers, resulting in gender imbalances in industries such as the electronic processing industry [27, 28]. Many rural mothers participate in China’s urban labor market [27, 29] and regularly circulate between rural villages and the cities [30, 31]. Consequently, for rural migrant working mothers in China, the decision regarding place of delivery is no longer between a hospital and home, but the choice between rural hometowns and urban areas.

In this study, rural migrant working mothers are defined as mothers “who have at least one child under 18 years of age, who have left the location of their agricultural hukou (household registration) for new work (employment or to start a business) in another location for a duration of at least one month” [32]. Generally, this research aims to analyze the childbirth strategies adopted by rural migrant working mothers. It specifically focuses on the choice between giving birth in their rural hometowns or urban areas. It also delves into the influence of various factors on this decision, including sociodemographic characteristics, child-related factors, migration-specific variables, health records and health education received in the host community. The Pearl River Delta (PRD) in southern China was selected due to its significance as one of China’s prominent special economic zones and is a major destination for rural migrant working mothers [27, 29]. By examining the childbirth strategies of rural migrant working mothers, the study could provide vital insights for health policy planning, particularly in urban areas where health facilities might be overwhelmed by the surge of migrant mothers. Moreover, it could aid in the development of tailored policies to enhance maternal and child health services.

## Methods and materials

### Study area: the Pearl river delta, China

The PRD, located in southern China, comprises nine cities within Guangdong province: Guangzhou, Shenzhen, Zhuhai, Foshan, Dongguan, Zhongshan, Huizhou, Jiangmen, and Zhaoqing. Since the implementation of economic reforms in 1978, the PRD has been at the forefront of China’s economic development, attracting substantial foreign investment and undergoing swift industrialization [33]. Not only is the PRD home to multinational manufacturing powerhouses like Foxconn and Flextronics, which are known for their labor-intensive and export-driven operations, but it also hosts rapidly growing domestic companies. Over the past decades, companies such as Huawei and Tencent have flourished, particularly in the production of communication technology-related products and services. China’s population and landscape have undergone significant transformations due to dual-track urbanization [34, 35]. At the end of 2022, the resident population (changzhu renkou) in the PRD was 78.29 million, though only 52.6% had local hukou (41.18 million) [36]. This suggests that the remaining 47.4% of the population may have been denied full citizenship rights, including the ability to enroll their children in local public schools since many of the citizenship rights are still associated with hukou in China.

### Data

The National Health Commission of China annually sponsored and coordinated nationwide cross-sectional surveys, known as the China Migrants Dynamic Survey (CMDS), to examine internal migrants from 2009 to 2018. The survey did not maintain a consistent data collection method each year. The 2016 CMDS was distinctive as it incorporated questions regarding migrants’ marital status, childbirth, childcare practices, health records and health education received in the host community. This unique dataset, capturing trends in childbirth location preferences among recent national migrants, offered a significant opportunity to investigate this crucial subject through a large, representative sample.

The survey was conducted in May 2016 by local Health Commissions and is accessible through an application process. The sample was selected using a stratified multi-stage random sampling method, employing a probability proportional to size (PPS) approach. Participants were residents aged 15 years or older who lacked a local hukou registration and had resided in the local communities for more than a month. Prior to participating in the survey, consent was obtained from all participants.

**Fig. 1:**
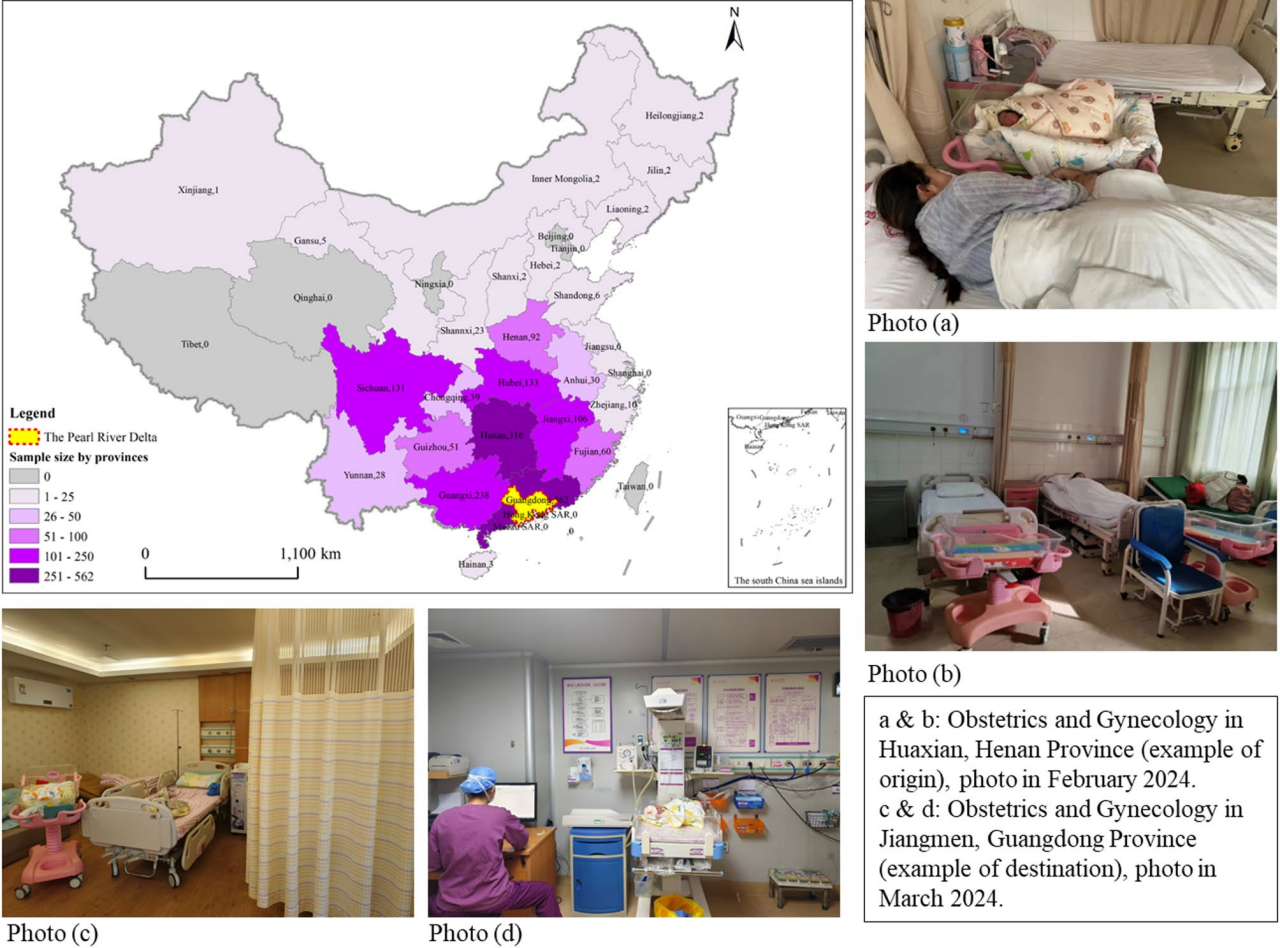
The number of samples of rural migrant working mothers per province of origin

The CMDS data sample used in this study encompassed rural migrant working mothers who fulfilled the following criteria: (i) being mothers who had at least one child under 18 years old; (ii) maintained an agricultural hukou registered outside of the PRD; and (iii) had been employed or engaged in business in any of the nine cities within the PRD for at least a month. A total of 1852 data points were collected to analyze the childcare strategies of rural migrant working mothers in the PRD. These 1852 rural migrants originated from 25 provinces in China (Fig. 1). Approximately 30.3% (562 of 1852) were intra-provincial migrants (originating from a non-PRD area within Guangdong province), and 69.7% (1290 out of 1852) were inter-provincial migrants (originating from outside Guangdong Province). The five most common provinces of origin for inter-provincial migrants were Hunan (17.1%, 316 out of 1852), Guangxi (12.0%, 238 out of 1852), Hubei (7.2%, 133 out of 1852), Sichuan (7.1%, 131 out of 1852), and Jiangxi (5.7%, 106 out of 1852). The identical dataset was utilized in another study focusing on childcare strategies among rural migrant workers in urban areas [37]. In addition to the sample distribution according to migrants’ origins, Fig. 1 also included photos of the physical environments of obstetrics and gynecology in Huaxian, Henan (an example of origin) and Jiangmen, Guangdong (an example of destination).

### Measurement

#### Dependent variable: place of delivery

Place of delivery was divided into two categories: rural hometowns (numerically denoted as “0”) and urban areas (numerically denoted as “1”). If a migrant working mother had more than one child, the information of the youngest child was used to simply data analysis and modeling, which also adhered to the inclusion criteria.

*Independent variables*.

i) Sociodemographic factors: The list of individual sociodemographic factors studied included age, education, type of job (employed or self-employed), individual and household income in the preceding month.
ii) Characteristics of the child: The characteristics included gender, age, and total number of older siblings (“0” meant the included case was the only kid in the family). In families with two or more children, the study selected the youngest child to identify the participants’ childbirth strategy. This approach was chosen to streamline the modeling and calculations.
iii) Migration-specific variables: In this study, rural migrant working mothers were categorized as inter-provincial or intra-provincial migrants, depending on whether their migration involved crossing a provincial-level administrative border. For inter-provincial migrants, the distance between their home province and their current city in the PRD was calculated using Google Maps. Since the city-level origin data for migrants was unavailable, an estimated average distance of 300 km was used for intra-provincial migrants, representing the distance between their native provinces and destination in the PRD. Furthermore, duration as a migrant worker was measured by the length of time in years a rural mother had worked as a migrant worker, which was also named migration duration.
iv) Health records and health education received in the host community: The study took into account whether participants had established a local resident health record and received health education in domains commonly addressed in the Chinese context. This specialized health education encompassed a variety of topics, including occupational diseases, HIV and sexually transmitted diseases/acquired immunodeficiency syndrome (STD/AIDS), reproductive health and contraception/eugenics, tuberculosis, smoking control, mental health, chronic diseases and nutrition.

### Analytical strategy

Initially, we produced descriptive statistics for the rural migrant working mother participants. Following this, we conducted an analysis of their place of delivery and scrutinized the variations in sociodemographic elements, child characteristics, migration-specific aspects, health records and health education received in the host community between two groups of mothers (those who gave birth in their rural hometowns or current urban areas).

In the third phase, we devised multivariate logistic regression models to investigate the influence of different variables on rural migrant working mothers’ delivery in urban areas. We utilized the following conceptual framework:

$$\begin{aligned}Log\left( {\frac{{{P_{Delivery\,in\,urban\,areas}}}}{{1 - {P_{Delivery\,in\,urban\,areas}}}}} \right)\,= &\,{\beta _0}\, + \,{\beta _1}\, \\ &\times \,va{r_1}\, + \, \ldots \, + \,{\beta _n}\,  \times \,va{r_n}\end{aligned}$$

In this framework, the variables var_1_ to var_n_ represent (a) sociodemographic factors (including age, education, job type, individual and household income); (b) child characteristics (including gender, age, and the total number of older siblings); (c) migration-specific data (including intra-provincial or inter-provincial migration, distance between native province and current city in the PRD, and duration as a migrant); and (d) health records and health education received in the host community (including the establishment of a local resident health record and health education on occupational diseases, STD/AIDS, reproduction and contraception/eugenics, tuberculosis, smoking control, mental health, chronic diseases and nutrition).

In addition to the initial four individual logistic regression models that encompassed four distinct categories of independent variables (Model 1 to 4), a comprehensive second-stage logistic regression model was formulated (Model 5). This model integrated all variables and was designed to self-adjust for the influential impacts of factors across categories. All logistic regression analyses reported adjusted odds ratios (OR) with 95% confidence intervals (CI), estimating the unique effect each factor had on the childbirth location of rural migrant working mothers. Moreover, the outcomes of the first-stage models were compared with the second-stage model to determine if the OR and the 95% CI of the latter were higher or lower than the former.

For each logistic regression model, the Nagelkerke R Square and Hosmer-Lemeshow test results were included in the model summary. Small values accompanied by large p-values in the Hosmer-Lemeshow test suggested a good model fit to the data. Conversely, large values with p-values below 0.05 indicated a poor fit. All analyses were executed using SPSS software (version 28.0) with a two-tailed statistical significance set at α = 0.05.

## Results

### Comparative analysis

In the sample of 1852 rural migrant working mothers, 63.7% (or 1180 mothers) chose to give birth in their rural hometowns. Conversely, 36.3% (or 672 mothers) opted for urban areas to deliver their newborns. The comparative analysis of birthplace choice among rural migrant working mothers reveals significant differences in sociodemographic factors, characteristics of the child, migration-specific variables, health records and health education received in the host community.

In terms of sociodemographic factors, rural migrant working mothers who gave birth in urban areas were slightly younger (average age of 32.1 years) compared to those in rural hometowns (average age of 33.9 years) (F(1) = 36.291, *p* < 0.001). There was also a significant variation in education levels, with a larger percentage of mothers giving birth in urban areas possessing a senior secondary (28.3%) or postsecondary degree (10.4%) in comparison to those who gave birth in rural hometowns (21.6% for senior secondary; 6.4% for postsecondary). The type of employment also differed, with a higher proportion of self-employed mothers giving birth in urban areas (45.9%) compared to those in rural hometowns (39.8%) (χ2(1) = 6.550, *p* < 0.05). Overall, rural migrant working mothers who gave birth in urban areas reported higher individual and household income than their counterparts who gave birth in rural hometowns (Table 1).

Child characteristics also differed. There were more girls born in urban areas (43.0%) than in rural hometowns (35.7%, χ2(1) = 9.726, *p* < 0.01). Children born in urban areas were generally younger, with an average age of 4.30 years, compared to those born in rural hometowns, who had an average age of 6.95 years (F(1) = 167.953, *p* < 0.001). They also had more siblings on average (mean = 0.72, SD = 0.70) than those born in rural hometowns (mean = 0.64, SD = 0.66).

The comparative analysis of migration-specific variables revealed that a larger proportion of mothers giving birth in urban areas were intra-provincial migrants (36.3%) as compared to those in rural hometowns (26.9%). On average, rural migrant working mothers traveled 774 km (SD = 493) from their rural hometowns to current cities in the PRD. However, there was no significant difference in the travel distance between mothers who gave birth in urban areas and those who did so in rural hometowns (F(1) = 2.133, *p* = 0.144). The duration as a migrant worker was typically longer for mothers who gave birth in urban areas, with an average duration of 6.89 years, as opposed to those in rural hometowns, who had an average duration of 4.46 years (F(1) = 17.165, *p* < 0.001).

Regarding health education received in the host community, a higher percentage of mothers who gave birth in urban areas received education on reproduction and contraception/eugenics (83.2%) compared to those in rural hometowns (76.6%) (χ2 (1) = 11.153, *p* < 0.001). Education on nutrition was also more prevalent among mothers who gave birth in urban areas (45.5%) than those in rural hometowns (38.4%). However, no significant disparities were observed in the establishment of local resident health records and receiving health education on occupational diseases, STD/AIDS, tuberculosis, smoking control, mental health, and chronic diseases between the two groups of mothers.

**Table 1:** Comparative analysis of place of delivery (rural hometown vs. urban area) among rural migrant working mothers (*N* = 1852)

Independent variable		Total samples No. (%)/ Mean (SD)	Rural hometowns No. (%)/ Mean (SD)	Urban areas No. (%)/ Mean (SD)	Chi-squared test / ANOVA Test^#^
**Sociodemographic factors**	Age (years)	33.3 (6.38)	33.9 (6.50)	32.1 (6.00)	F(1) = 36.291, *p* < 0.001
	Education				
	*Primary or below*	200 (10.8%)	144 (12.2%)	56 (8.3%)	χ2(3) = 26.376, *p* < 0.001
	*Junior secondary*	1062 (57.3%)	706 (59.8%)	356 (53.0%)	
	*Senior secondary*	445 (24.0%)	255 (21.6%)	190 (28.3%)	
	*Postsecondary*	145 (7.8%)	75 (6.4%)	70 (10.4%)	
	Type of job				
	*Employee*	1063 (58.0%)	703 (60.2%)	360 (54.1%)	χ2(1) = 6.550, *p* < 0.05
	*Self-employed*	771 (42.0%)	465 (39.8%)	306 (45.9%)	
	Individual income (RMB/month)	3897 (3950)	3672 (3171)	4291 (5103)	F(1) = 10.563, *p* < 0.001
	Log_10_(Individual income)	3.51 (0.24)	3.50 (0.21)	3.53 (0.27)	F(1) = 6.552, *p* < 0.05
	Household income (RMB/month)	8544 (6668)	8032 (5202)	9442 (8593)	F(1) = 19.330, *p* < 0.001
	Log_10_(Household income)	3.87 (0.21)	3.85 (0.20)	3.90 (0.22)	F(1) = 23.582, *p* < 0.001
**Characteristics of the child**	Gender				
	*Boy*	1142 (61.7%)	759 (64.3%)	383 (57.0%)	χ2(1) = 9.726, *p* < 0.01
	*Girl*	710 (38.3%)	421 (35.7%)	289 (43.0%)	
	Age of the child (years)	5.99 (4.41)	6.95 (4.58)	4.30 (3.52)	F(1) = 167.953, *p* < 0.001
	The number of elder sibling(s)	0.67 (0.68)	0.64 (0.66)	0.72 (0.70)	F(1) = 7.224, *p* < 0.01
**Migration-specific variables**	Type of migrant				
	*Intra-provincial*	562 (30.3%)	318 (26.9%)	244 (36.3%)	χ2(1) = 17.748, *p* < 0.001
	*Inter-provincial*	1290 (69.7%)	862 (73.1%)	428 (63.7%)	
	Distance (kilometers)	774 (493)	787 (473)	752 (526)	F(1) = 2.133, *p* = 0.144
	Log_10_(Distance)	2.81 (0.26)	2.82 (0.26)	2.79 (0.28)	F(1) = 7.470, *p* < 0.001
	Duration as a migrant worker (years)	8.01 (6.05)	4.46 (4.18)	6.89 (5.18)	F(1) = 17.165, *p* < 0.001
**Health records and health education received in the host community**	Established a local resident health record				
	*No*	972 (52.5%)	629 (53.3%)	343 (51.0%)	χ2(1) = 0.880, *p* = 0.348
	*Yes*	880 (47.5)	551 (46.7%)	329 (49.0%)	
	Occupational diseases				
	*No*	1143 (61.7%)	719 (60.9%)	424 (63.1%)	χ2(1) = 0.848, *p* = 0.357
	*Yes*	709 (38.3%)	461 (39.1%)	248 (36.9%)	
	STD/AIDS				
	*No*	977(52.8%)	628 (53.2%)	349 (51.9%)	χ2(1) = 0.284, *p* = 0.594
	*Yes*	875(47.2%)	552 (46.8%)	323 (48.1%)	
	Reproduction and contraception/ eugenics				
	*No*	389(21.0%)	274 (23.4%)	113 (16.8%)	χ2(1) = 11.153, *p* < 0.001
	*Yes*	1463(79.0%)	904 (76.6%)	559 (83.2%)	
	Tuberculosis				
	*No*	1519 (82.0%)	971 (82.3%)	548 (81.5%)	χ2(1) = 0.159, *p* = 0.690
	*Yes*	333 (18.0%)	209 (17.7%)	124 (18.5%)	
	Smoking control				
	*No*	1175 (63.4%)	757 (64.2%)	418 (62.2%)	χ2(1) = 0.702, *p* = 0.402
	*Yes*	677 (36.6%)	423 (35.8%)	254 (37.8%)	
	Mental health				
	*No*	1594 (86.1%)	1025 (86.9%)	569 (84.7%)	χ2(1) = 1.715, *p* = 0.190
	*Yes*	258 (13.9%)	155 (13.1%)	103 (15.3%)	
	Chronic disease				
	*No*	1392 (75.2%)	890 (75.4%)	502 (74.7%)	χ2(1) = 0.119, *p* = 0.730
	*Yes*	460 (24.8%)	290 (75.4%)	170 (25.3%)	
	Nutrition				
	*No*	1093 (59.0%)	727 (61.6%)	366 (54.5%)	χ2(1) = 9.039, *p* < 0.01
	*Yes*	759 (41.0%)	453 (38.4%)	306 (45.5%)	

Note: #, significant results in the Chi-squared test/ ANOVA test (*p* < 0.05) indicated a statistical difference among the two studied sub-groups; Chi-square tests were conducted for the categorical variables, including education, types of job, gender of child, type of migrant and all health education indicators; ANOVA tests were conducted for the continues variables, including age, individual income, log10(individual income), household income, log10(household income), age of the child, number of elder siblings, distance, log10(distance) and duration as a migrant worker

### Regression analysis of rural migrant working mothers’ place of delivery

#### (1) association with sociodemographic factors

Age had complex impacts on rural migrant working mothers’ place of delivery since it was negatively associated with urban delivery in the first-stage model 1 (OR = 0.960, 95% CI: 0.944–0.976), but not significant in the second-stage model 5 (OR = 1.002, 95% CI: 0.974–1.030) when the characteristics of the child, spatial and temporal migration variables and health education were held constant (Table 2). Higher education levels showed a positive correlation with the decision to give birth in urban areas. Specifically, having a postsecondary education degree made rural migrant working mothers 45.7% (OR = 1.457, 95% CI: 1.014–1.605 in Model 5) to 76.7% (OR = 1.767, 95% CI: 1.089–2.867 in Model 1) more likely to adopt urban delivery. In addition, being self-employed increased the likelihood of delivery in urban areas by 27.6% (OR = 1.276, 95% CI: 1.014–1.605 in Model 5) to 38.6% (OR = 1.386, 95% CI: 1.131–1.698 in Model 1). Household income also showed a positive correlation, while individual income was not significantly associated with place of delivery in both Model 1 and Model 5. An increase of one unit in household income on a log10 scale increased a mother’s likelihood of adopting the urban delivery by 135.0% (OR = 2.350, 95% CI: 1.253–4.409 in Model 5) to 155.8% (OR = 2.558, 95% CI: 1.437–4.551 in Model 1). It’s observed that the effect of all sociodemographic factors decreased slightly when including child characteristics, migration-specific variables and health education in Model 5.

#### (2) association with the characteristics of the child

The age of the child (meaning the birth year) was negatively associated with the rural migrant working mothers’ place of delivery. In particular, each additional year of the child’s age (i.e., each earlier birth year) corresponded to a 15.6% reduction in the likelihood of choosing urban areas for childbirth when only the child’s characteristics were taken into account (OR = 0.844, 95% CI: 0.821–0.867 in Model 2). This reduction increased to 20.5% when sociodemographic factors, migration-specific variables and health education were also factored in (OR = 0.795, 95% CI: 0.763–0.829 in Model 5). Conversely, the number of older siblings positively impacted giving birth in urban areas. One additional older sibling increased the probability of adopting the urban delivery by 38.0% (OR = 1.380, 95% CI: 1.150–1.656 in Model 5) to 47.3% (OR = 1.473, 95% CI: 1.266–1.713 in Model 2). The child’s gender, however, did not significantly influence the place of delivery in either Model 2 or Model 5.

#### (3) association with migration-specific variables

Inter-provincial rural migrant working mothers were 46.8% (OR = 0.532, 95% CI: 0.366–0.774 in Model 3) to 50.1% (OR = 0.499, 95% CI: 0.330–0.754 in Model 5) less likely to give birth in urban areas. The duration as a migrant worker was positively associated with the decision of where to deliver. Specifically, every additional year of migration duration was associated with a 3.2% increased probability of choosing urban areas for childbirth when only considering the migrant-specific variables (OR = 1.032, 95% CI: 1.016–1.048 in Model 3) and it was increased to 9.8% when the other factors were included in the models (OR = 1.098, 95% CI: 1.075–1.121 in Model 5). While migration distance was not a significant factor when considering only migration-specific (OR = 1.544, 95% CI: 0.803–2.968 in Model 3), it became a positive factor when sociodemographic factors, the characteristics of the child and health education were also taken into account (OR = 2.462, 95% CI: 1.189–5.096 in Model 5). In this context, an increase in the standard deviation of Log10(Distance) corresponded to a 9.8% increase in the likelihood that the children were born in urban areas (OR = 1.098, 95% CI: 1.075–1.121 in Model 3). It’s also observed that the effect of all migrant-specific factors exhibited a slight increase when including sociodemographic factors, child characteristics and health education in Model 5.

#### (4) association with Spatial and Temporal migration variables

Health education received in the host community also influenced the decision of where to deliver. Mothers who received education on reproduction and contraception/eugenics, and nutrition were more likely to give birth in urban areas. Specifically, receiving health education on reproduction and contraception/eugenics led to a 47.8% (OR = 1.478, 95% CI: 1.132–1.931 in Model 4) to 58.3% (OR = 1.583, 95% CI: 1.178–2.128 in Model 5) increase in the likelihood of rural migrant working mothers’ delivery in urban areas. Meanwhile, receiving health education on nutrition increased the same likelihood by 31.0% (OR = 1.310, 95% CI: 1.053–1.631 in Model 4) to 35.4% (OR = 1.354, 95% CI: 1.062–1.752 in Model 5). Established a local resident health record and the other types of health education, including occupational diseases, STD/AIDS, tuberculosis, smoking control, mental health, and chronic diseases, were not significant influencing factors.

Overall, the model summary indicates that the second-stage model 5 explains more variance (Nagelkerke R Square = 0.236) than the first-stage models (Table 2). The Hosmer-Lemeshow Test shows that all models have a good fit (*p* > 0.05).

**Table 2:** Logistic regression analysis of place of delivery among rural migrant working mothers (*N* = 1852; 0 = rural hometown; 1 = urban area)

Name of independent variable		First-stage				Second-stage
		Model 1	Model 2	Model 3	Model 4	Model 5
**Sociodemographic factors**	Age (years)	0.960, *p* < 0.001 [0.944, 0.976]				1.002, *p =* 0. [0.974, 1.030]
	Education					
	*Junior secondary*	1.045, *p* = 0.804 [0.737, 1.481]				0.094, *p* = 0.760 [0.638, 1.388]
	*Senior secondary*	1.455, *p* = 0.056 [0.990, 2.137]				1.345, *p* = 0.179 [0.873, 2.071]
	*Postsecondary*	1.767, *p* < 0.05 [1.089, 2.867]				1.457, *p* < 0. [1.014, 1.605]
	Type of job (self-employed)	1.386, *p* < 0.01 [1.131, 1.698]				1.276, *p* < 0.05 [1.014, 1.605]
	Log_10_(Individual income)	0.783, *p* = 0.333 [0.487, 1.284]				0.736, *p* = 0.263 [0.430, 1.259]
	Log_10_(Household income)	2.558, *p* < 0.01 [1.437, 4.551]				2.350, *p* < 0.01 [1.253, 4.409]
**Characteristics of the child**	Gender (girl)		1.177, *p* = 0.120 [0.958, 1.444]			1.158, *p* = 0.187 [0.932, 1.438]
	Age of the child (years)		0.844, *p* < 0.001 [0.821, 0.867]			0.795, *p* < 0.001 [0.763, 0.829]
	The number of elder sibling(s)		1.473, *p* < 0.001 [1.266, 1.713]			1.380, *p* < 0.001 [1.150, 1.656]
**Migration-specific variables**	Type of migrant (inter-provincial)			0.532, *p* < 0.001 [0.366, 0.774]		0.499, *p* < 0.001 [0.330, 0.754]
	Log_10_(Distance)			1.544, *p* = 0.193 [0.803, 2.968]		2.462, *p* < 0.05 [1.189, 5.096]
	Duration as a migrant worker (years)			1.032, *p* < 0.001 [1.016, 1.048]		1.098, *p <* 0.001 [1.075, 1.121]
**Health records and health education received in the host community**	Established a local resident health record (yes)				0.963, *p* = 0.708 [0.790, 1.173]	1.111, *p* = 0.348 [0.891, 1.386]
	Occupational diseases (yes)				0.814, *p* = 0.087 [0.643, 1.030]	0.831, *p* = 0.170 [0.638, 1.082]
	STD/AIDS (yes)				0.916, *p* = 0.449 [0.730, 1.150]	0.929, *p* = 0.566 [0.724, 1.193]
	Reproduction and contraception/ eugenics (yes)				1.478, *p* < 0.01 [1.132, 1.931]	1.583, *p* < 0.01 [1.178, 2.128]
	Tuberculosis (yes)				0.996, *p* = 0.984 [0.697, 1.424]	1.131, *p* = 0.545 [0.760, 1.683]
	Smoking control (yes)				0.995, *p* = 0.964 [0.792, 1.250]	0.953, *p* = 0.712 [0.740, 1.228]
	Mental health (yes)				1.169, *p* = 0.403 [0.811, 1.685]	1.209, *p* = 0.363 [0.803, 1.820]
	Chronic disease (yes)				0.936, *p* = 0.646 [0.705, 1.243]	0.844, *p* = 0.289 [0.617, 1.154]
	Nutrition (yes)				1.310, *p* < 0.05 [1.053, 1.631]	1.354, *p* < 0.05 [1.062, 1.725]
**Model summary**	Nagelkerke R Square	0.055	0.138	0.026	0.016	0.236
	Hosmer-Lemeshow Test	χ2(8) = 5.299, *p* = 0.725	χ2(8) = 12.228, *p* = 0.141	χ2(8) = 12.919, *p* = 0.115	χ2(8) = 10.221, *p* = 0.250	χ2(8) = 4.305, *p* = 0.829

Note: Adjusted odds ratios (OR) with p-value and 95% confidence intervals (CI) were reported for each independent variable. The dependent variable was the place of delivery among rural migrant working mothers (0 = rural hometown; 1 = urban area). Model 1 included only sociodemographic factors as independent variables; Model 2 included only characteristics of the child; Model 3 included only migration-specific variables; Model 4 included only health records and health education received in the host community as independent variables. Model 5 integrated all variables and was designed to self-adjust for the influential impacts of factors across categories

## Discussion

### Rural migrant working mothers’ birthplace choice in China

A growing number of rural mothers are participating in the urban labor market, which is a stark contrast to the situation 20 years ago when few married women participated in rural-to-urban migration [38]. In this study, rural mothers journeyed an average of over 700 km from their rural hometowns to the PRD, primarily to financially support their families. A significant majority of these rural migrant working mothers (63.7%, or 1180 out of 1852) opted to return to their hometowns for childbirth, while a smaller proportion (36.6%, or 672 out of 1852) chose to give birth in urban areas. The choice of childbirth location was influenced by various factors including women’s sociodemographics, characteristics of the child, migration and exposure to health education. Factors that positively influenced a rural migrant working mother’s decision to deliver in urban areas included being self-employed, having postsecondary education, higher household income (measured on a log-10 scale), more children, longer migration duration, receiving health education on reproduction, contraception/eugenics, and nutrition. Meanwhile, inter-provincial migration and earlier birth year negatively influenced rural migrant working mothers’ giving birth in urban areas.

In post-reform China, many rural families require dual-income earners to meet their needs, leading rural migrant working mothers to juggle dual roles as caregivers and income providers [39]. However, the scarcity of affordable childcare facilities often poses challenges for these women seeking full-time employment in the labor market [39]. As a result, many of these mothers opt for alternative employment structures, such as self-employment, which offers the flexibility needed to balance family responsibilities with work [40]. Small-scale businesses, including roles like street vendors or hawkers, have emerged as favored self-employment options for many rural migrant workers in urban China [41]. These mothers, with self-employed, may find it challenging to leave their businesses unattended for a long period. Giving birth in urban areas where they work allows them to continue their business and manage their business more effectively without significant breaks.

Moreover, rural migrant working mothers with a postsecondary education level are more likely to give birth in urban areas than those with lower education levels. Higher education often correlates with a better understanding of healthcare standards and the importance of access to quality medical services [42]. Educated mothers are more likely to be aware of the benefits of advanced medical facilities available in urban areas and therefore choose to give birth there to ensure better healthcare for themselves and their newborns. Mothers with higher education levels are also often more focused on the long-term educational and developmental benefits for their children [28]. Knowing that urban areas typically offer better educational facilities and extracurricular opportunities [30], they might choose to give birth and raise their children in these environments.

Furthermore, rural migrant working mothers with higher household incomes are more likely to choose urban areas for childbirth, whereas individual income does not significantly influence this decision. When studying working mothers, it is crucial to consider both individual and household income, as they can differently impact behaviors and decisions. For example, a study in China found that rural migrant working mothers with higher individual incomes were more likely to leave their children behind in the countryside, while those with higher household incomes were more likely to move to urban areas with their children [37]. This distinction highlights the varying influences of individual versus household economic resources on family migration decisions. In our research, rural migrant working mothers with higher household income reported higher possibility to deliver in urban areas. Urban healthcare facilities often offer better services and technologies (Fig. 1) but at a higher cost [31]. Higher household income makes it more feasible for the families of rural migrant workers to afford these premium health services [43], ensuring a safer and more comfortable birthing experience for migrant mothers. A higher income also generally enables to secure better housing for rural migrants in urban environments and many of these individuals do not need to rely on the dormitories provided by their employers [32]. This stability is significant when considering where to give birth, as it influences factors like proximity to quality healthcare and overall living conditions.

The decision to give birth in urban areas is also influenced by the age of the child and the number of older siblings. Specifically, each earlier birth year, meaning each additional year of the child’s age corresponds to a 15.6–20.5% reduction in the likelihood of choosing urban areas for childbirth. This suggests a notable increase in the number of rural migrant working mothers choosing urban areas for childbirth in recent years compared to previous periods. Over the years, urban areas tend to have larger migrant communities [44], providing a support network for rural migrant working mothers during pregnancy and childbirth. These communities can offer emotional support, practical advice, and assistance during the transition to motherhood. Our data also indicates that having one additional older sibling increases the likelihood of adopting an urban delivery by 38.0–47.3%. Rural migrant working mothers with more than one child are likely more aware of the potential complications and needs that can arise during childbirth [45] and may prefer the medical services available in cities to ensure the health and safety of both the newborn and the rest of the family. Additionally, these mothers gain more experience with each subsequent child, reducing their reliance on family support from their rural hometowns and increasing their confidence in delivering in urban areas.

In addition, our research also uncovers that crossing provincial administrative boundaries has a significant influence on the childbirth strategies of rural migrant working mothers, whereas the distance migrated did not have a significant impact. Specifically, inter-provincial rural migrant working mothers are found to have a 46.8–50.1% lower likelihood of opting for urban delivery compared to their intra-provincial counterparts. Moving to a different province often means encountering different cultural norms and potentially different dialects [46]. When compared to inter-provincial counterparts, intra-provincial rural migrant working mothers may have access to existing social support networks within their province, such as family members, friends, or fellow migrants who can provide assistance and guidance during pregnancy and childbirth. Rural migrant working mothers in the PRD with a longer temporal duration of migration reported a higher likelihood of giving birth in urban areas. This finding is consistent with similar research conducted using data collected in 2014 [47]. Over time, rural migrant working mothers may become more adapted to the urban lifestyle, culture, and environment, making them more comfortable with giving birth in the city. The longer temporal duration of migration also often means that the migrants have achieved a certain level of financial stability, making it feasible for them to afford healthcare services in urban areas.

Finally, many migrant women did not receive adequate antenatal care and initiated antenatal care especially later than the optimal first 12 weeks of pregnancy [48]. Furthermore, research has shown that 12.6% of rural migrant women in China did not undergo any examination during the first trimester, while 27.6% had less than five prenatal visits during their latest pregnancy according to the data collected in 2014 [45]. Receiving health education enhances the understanding of rural migrant working mothers about reproductive health, family planning, nutrition and childcare. This knowledge can help them make informed decisions about where to give birth. As a result, they may opt for urban areas due to superior medical facilities and the long-term benefits of raising children in cities.

### Limitations and future research

This study aimed to enrich our understanding of the factors influencing the childbirth location choices of rural migrant working mothers. However, due to data availability constraints, our analysis only included information on the youngest child if a rural mother had two or more children, which was the case for over half of rural working migrant mothers. Childbirth strategies may be more diverse for families with two or more children, as some of the older children might be born in rural hometowns while the younger children were born in urban areas. Moreover, our study only encompassed rural migrant mothers involved in income-generating activities. As such, our findings might primarily reflect the behaviors of income-earning rural migrant mothers. Those who had exited the labor market for any duration were not represented in our sample. In addition to individual factors (e.g., sociodemographic factors, migration-specific variables and health education), childbirth location choice can be influenced by broader determinants such as medical insurance policies, maternity leave provisions, accessibility and quality of maternal healthcare services, cultural norms and economic conditions in both migrants working mothers’ household registration areas and workplaces. Future research may seek to incorporate such macro-level data to provide a more comprehensive understanding of the determinants influencing delivery place choices among migrant mothers. Furthermore, our study established a significant baseline by using the quantitative data collected in 2016 through a national survey to understand rural migrant working mothers’ places of delivery in the PRD. This is important for evaluating the effectiveness of subsequent relevant healthcare reforms targeting migrant populations. We recommend that future research incorporate qualitative methodologies and contextual analysis to gain a deeper understanding of the childbirth decisions made by rural migrant working mothers across a broader geographical spectrum in China.

## Conclusions

This study deepens the understanding of the place of delivery among rural migrant working mothers. The selection of place of delivery is not solely an individual choice but is influenced by various factors including the mothers’ type of employment, education level, number of children, the time and place of their migration, and health education. The findings from this study will be instrumental for upcoming policy research and can offer evidence-based recommendations for policymakers addressing internal rural-to-urban migration. It has become increasingly important for both destination and origin governments to provide necessary services throughout all stages of childbirth for rural migrant working mothers, as a strategy to address low fertility rates. Going forward, additional research should be undertaken to establish an evidence-based framework that explores the relationship between migration and childbirth for the integrated health and wellbeing of rural migrant working women and their families.

## Data Availability

The data analyzed in this study may be requested via the Migrant Population Service Center, National Health Commission of China.
